# A New Model for the Predicting the Risk of Preeclampsia in Twin Pregnancy

**DOI:** 10.3389/fphys.2022.850149

**Published:** 2022-04-08

**Authors:** Qing Han, Shuisen Zheng, Rongxin Chen, Huale Zhang, Jianying Yan

**Affiliations:** Department of Obstetrics, Fujian Maternity and Child Health Hospital College of Clinical Medicine for Obstetrics & Gynecology and Pediatrics, Fujian Medical University, Fuzhou, China

**Keywords:** twins, preeclampsia, least absolute shrinkage and selector operator regression, nomogram, obstetrics

## Abstract

**Objective:**

We aimed to develop an effective nomogram model for predicting the risk of preeclampsia in twin pregnancies.

**Methods:**

The study was a retrospective cohort study of women pregnant with twins who attended antenatal care and labored between January 2015 and December 2020 at the Fujian Maternity and Child Health Hospital, China. We extracted maternal demographic data and clinical characteristics. Then we performed the least absolute shrinkage and selection operator regression combined with clinical significance to screen variables. Thereafter, multivariate logistic regression was used to construct a nomogram that predicted the risk of preeclampsia in twin pregnancies. Finally, the nomogram was validated using C-statistics (C-index) and calibration curves.

**Results:**

A total of 2,469 women with twin pregnancies were included, of whom 325 (13.16%) had preeclampsia. Multivariate logistic regression models revealed that serum creatinine, uric acid, mean platelet volume, high-density lipoprotein, lactate dehydrogenase, fibrinogen, primiparity, pre-pregnancy body mass index, and regular prenatal were independently associated with preeclampsia in twin pregnancies. The constructed predictive model exhibited a good discrimination and predictive ability for preeclampsia in twin pregnancies (concordance index 0.821).

**Conclusion:**

The model for the prediction of preeclampsia in twin pregnancies has high accuracy and specificity. It can be used to assess the risk of preeclampsia in twin pregnancies.

## Introduction

Preeclampsia is a unique complication of pregnancy and occurs in 5–7% of all pregnancies. It is the leading cause of maternal and fetal perinatal morbidity and mortality (Rana et al., 2019). The etiology and pathogenesis of preeclampsia remain unclear (Ives et al., 2020). Besides, there is no effective treatment for preeclampsia in addition to the termination of pregnancy. Therefore, it is important to strengthen the screening and prediction of high-risk groups before delivery. With the development of assisted reproductive technology, the incidence of twin pregnancy is increasing yearly (American College of Obstetricians and Gynecologists, 2021). Clinical studies have shown that the incidence of preeclampsia is two to three times higher in twin than in singleton pregnancies (Baha et al., 2000; Wang et al., 2021). However, in recent years, research on the risk prediction of preeclampsia is mostly limited to singleton pregnancies. Maternal serum makers such as coagulation function and liver function are considered to be closely related to preeclampsia (Duan et al., 2020). In addition, many studies have shown that primiparas and pregnant women with a high pregestational body mass index are more likely to develop preeclampsia (Yang et al., 2021). But it is unclear whether these predictors in singletons equally apply to twin pregnancies. Therefore, we conducted a retrospective cohort study to explore the high-risk factors associated with preeclampsia in twin pregnancies and built a clinical prediction model to guide early interventions and improve pregnancy outcomes.

## Materials and Methods

### Research Object

**FIGURE 1:**
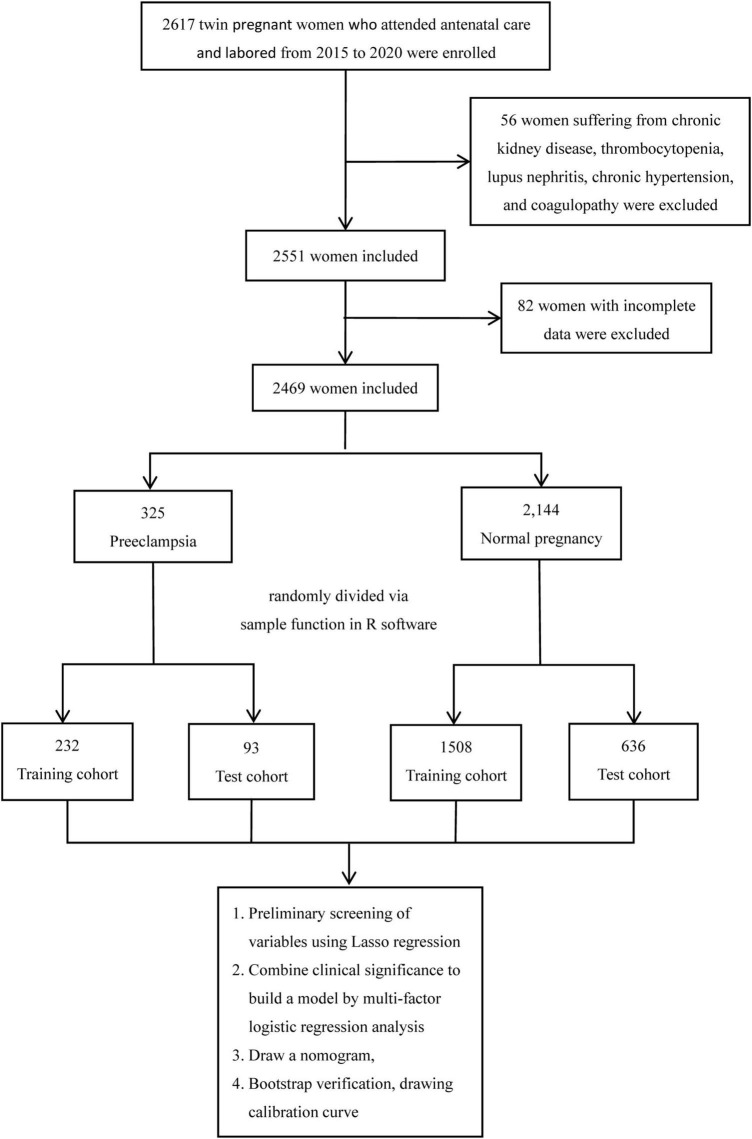
Flowchart of participant selection for the study cohort.

We performed a population-based retrospective cohort study on women with twin pregnancies who attended antenatal care and labored at the Fujian Maternity and Child Health Hospital between January 2015 and December 2020. We excluded women with the following conditions: thrombocytopenia, chronic kidney disease, lupus nephritis, coagulopathy, gestational hypertension and chronic hypertension. A total of 2,469 women with twin pregnancies were included in the study (Figure 1). According to clinical diagnosis, they were divided into a preeclampsia group (325 cases) and a normal pregnancy group (2,144 cases). None of the women in the normal pregnancy group were hypertensive.

The diagnostic criteria for preeclampsia were in reference to *Williams Obstetrics* (Cunningham, 2018): Systolic Blood Pressure (SBP) ≥ 140 mm Hg and/or diastolic blood pressure (DBP) ≥ 90 mm Hg on at least two occasions, 4 h apart, at first time after 20 weeks of pregnancy, combined with proteinuria of 300 mg in 24 h, or two readings of at least 2 Þ on dipstick analysis of midstream or catheter urine specimens if no 24-h collection was available. Whether the women was included in the preeclampsia group or the normal pregnancy group was decided after discussion by two or more attending obstetricians.

### Measurement Methods

Participants underwent routine blood tests when hospitalized for other indications, such as childbirth and abnormal fetal heart monitoring. All the blood tests (blood routine, liver function, kidney function, blood lipid parameters, and coagulation parameters) were performed before the clinical diagnosis of preeclampsia. We used an automatic hematology system XE-5000 (Sysmex Corporation, Japan) to measure peripheral blood variables.

### Procedures

The following data were collected from electronic medical records of the hospital: (1) basic information and medical history, including age, height, weight, body mass index (BMI), gravidity, parity, prenatal visits, chronicity, and *in vitro* fertilization–embryo transfer; (2) auxiliary examination findings, including routine blood parameters [red blood cell count, platelets, hematocrit, platelet distribution width, and mean platelet volume (MPV)], liver function [aspartate aminotransferase (AST), alanine aminotransferase (ALT), lactate dehydrogenase (LDH), and transglutaminase (GGT)], kidney function [serum creatinine (CREm) and uric acid (URIC)], blood lipid parameters [cholesterol, triglycerides (TG), apolipoprotein-b, high-density lipoprotein (HDL), and low-density lipoprotein (LDL)], and coagulation parameters [prothrombin time (PT), fibrin degradation products, thrombin time, D-dimer, fibrinogen (Fib), activated partial thromboplastin time (APPT), and international normalized ratio (INR)]; (3) maternal complications: gestational diabetes and premature rupture of membranes.

### Statistical Analysis

We compared the differences between the two groups using the independent sample *t*-test or non-parametric tests. Qualitative data are expressed as frequencies and percentages, and the Pearson’s Chi-squared test or Fisher’s exact test were conducted to analyze differences between groups. To screen and validate variables relate to preeclampsia, all women in the study were randomly divided into a training cohort and a test cohort. The training cohort (*n* = 1,740) account for 70% of the participants and the test cohort (*n* = 729) account for 30%. The least absolute shrinkage and selection operator (LASSO) regression with 10-fold cross-validation was used to select the most useful predictive variables *via* lse criteria in the training cohort, where “lse” refers to the largest lambda for which the mean squared percentage error (MPSE) is within one standard error of the minimal MSPE. Then Internal validation was performed, the receiver operating characteristic (ROC) analysis was performed (*via* the survival ROC package), and the area under the curve (AUC) was calculated to evaluate the discrimination in the test cohort. Thereafter, combining with clinical significance, we built the model with multivariate logistic regression. A nomogram of the combined models was formulated to visualize the efficiency of these models. The calibration curves and Harrell’s concordance index (C-Index) were used to assess the ability of various models to predict the risk of preeclampsia in twin pregnancies. Bootstrap re-sampling (1,000 re-samples) was used for plotting calibration curves. Quantitative data are presented as mean ± standard deviation, denoted as X ± SD or medians with interquartile range. We collected data for this study in Excel (Microsoft^®^ Excel^®^ 2010), and analyzed and graphed them using the SPSS™ Statistics v25.0 (IBM, Armonk, NY, United States) and R software (v3.6.2).

### Ethical Approval

This study obtained an approval from the ethical committee of the Fujian Maternity and Child Health Hospital. An informed written consent for the patients was not required because only information from unidentifiable patients was used.

## Results

### Baseline Characteristics

In total, 2,469 women with twin pregnancies were included, of whom 325 (13.16%) women had preeclampsia. There were no statistically significant differences in clinical characteristics between the training cohort (*n* = 1,740) and the test cohort (*n* = 729) (Table 1).

**TABLE 1:** Clinical characteristics of the training and test cohort.

	Training cohort (*n* = 1 740)	Test cohort (*n* = 729)	*P* value
Age (y)	29.9 ± 4.41^a^	30.12 ± 4.38^a^	0.286
Height (cm)	160.17 ± 4.99^a^	160.30 ± 5.17^a^	0.565
Pre-pregnancy weight (kg)	54.46 ± 14.35^a^	54.67 ± 8.56^a^	0.703
Pre-pregnancy BMI (kg/m^2^)	27.71 ± 6.46^a^	27.64 ± 3.27^a^	0.774
Gravidity	2.00 [1.00, 2.00]	2.00 [1.00, 2.00]	0.157
Parity	0.00 [0.00, 1.00]	0.00 [0.00, 1.00]	0.438
Primipara [*n* (%)]	881 (50.6)	393 (53.9)	0.149
Regular prenatal visits [*n* (%)]	1,217 (69.9)	537 (73.7)	0.07
IVF-ET [*n* (%)]	582 (33.4)	230 (31.6)	0.385
Chorionicity [*n* (%)]			0.642
Monochorionic	545 (31.3)	236 (32.4)	
Dichorionic	1,195 (68.7)	493 (67.6)	
GDM [*n* (%)]	1,357 (78.0)	548 (75.2)	0.142
PROM [*n* (%)]	430 (24.7)	164 (22.5)	0.261
Preeclampsia [*n* (%)]	232 (13.3)	93 (12.8)	0.699

*^a^Mean and standard deviation; BMI, body mass index; IVF-ET, in vitro fertilization- embryo transfer; GDM, gestational diabetes mellitus; PROM, premature rupture of membranes.*

We compared the general baseline data of the preeclampsia and the normal pregnancy groups and found there were no statistically significant differences in age, height, *in vitro* fertilization–embryo transfer, choriocarcinoma, and gestational diabetes between the two groups. The gestational age in the normal pregnancy group was greater than that in the preeclampsia group, but there was no statistical significance (*P* = 0.644). Both systolic pressure and diastolic pressure in the preeclampsia group was greater than in the normal pregnancy group (*P* < 0.001 and < 0.001, respectively). The proportion of primipara in the preeclampsia group was greater than in the normal pregnancy group (*P* = 0.006), while the proportion of pregnant women who received regular check-ups was significantly lower in the preeclampsia group than in the normal pregnancy group (*P* = 0.007). The incidence of premature rupture of membranes in the normal pregnancy group was higher than that in the preeclampsia group (*P* = 0.003). Similarly, the incidence of preterm premature rupture of membranes was higher in the normal pregnancy group compared with the preeclampsia group (*P* = 0.006). A comparison of the serological indicators in the two groups revealed that, in terms of renal function parameters, URIC and CREm were significantly higher in the preeclampsia group than in the normal pregnancy group, and the difference was statistically significant. As for liver function indicators, the differences in AST and LDH were statistically significant, while the differences in ALT and GGT between the two groups were not statistically significant. In terms of blood lipid indices, HDL was significantly lower in the preeclampsia group than in the normal pregnancy group (*P* < 0.001), but the difference in TG between the two groups was not statistically significant. Regarding coagulation indicators, the differences in fibrin degradation products, thrombin time, D-dimer, Fib, and APPT between the two groups were statistically significant, while the differences in PT and INR were not statistically significant. In terms of routine blood indicators, red blood cell count, platelet count, and hematocrit were lower in the preeclampsia group than in the normal pregnancy group, while MPV and platelet distribution width were higher in the preeclampsia group than in the normal pregnancy group, and the difference was statistically significant (Table 2).

**TABLE 2:** General baseline information.

	Normal pregnancy (*n* = 2,144)	Preeclampsia (*n* = 325)	*P* value
Age (y)	29.97 ± 4.40^a^	30.09 ± 4.64^a^	0.597
Height (cm)	160.18 ± 5.03^a^	160.43 ± 5.15^a^	0.396
Pre-pregnancy weight (kg)	54.24 ± 8.07^a^	56.34 ± 28.92^a^	0.006
Pre-pregnancy BMI (kg/m^2^)	22.94 ± 3.36^a^	23.70 ± 3.65^a^	<0.001
Gestational week	37.25 ± 58.36^a^	35.75 ± 2.06^a^	0.644
Systolic blood pressure (mmHg)	121.03 ± 9.52^a^	148.41 ± 15.89^a^	<0.001
Diastolic blood pressure (mmHg)	72.78 ± 8.48^a^	89.97 ± 10.77^a^	<0.001
Gravidity	2.00 [1.00, 2.00]	1.00 [1.00, 2.00]	0.014
Parity [*n* (%)]			0.018
0	1,014 (47.3%)	181 (55.7%)	
1	1,005 (46.9%)	127 (39.1%)	
≥ 2	125 (5.8%)	17 (5.2%)	
Primipara [*n* (%)]	1,014 (47.3%)	181 (55.7%)	0.006
Regular prenatal visits [*n* (%)]	1,544 (72.0%)	210 (64.6%)	0.007
IVF-ET [*n* (%)]	690 (32.2%)	122 (37.5%)	0.064
Chorionicity [*n* (%)]			0.391
Monochorionic	671 (31.3%)	110 (33.8%)	
Dichorionic	1,473 (68.7%)	215 (66.2%)	
GDM [*n* (%)]	491 (22.9%)	73 (22.5%)	0.916
PROM [*n* (%)]	538 (25.1%)	56 (17.2%)	0.003
PPROM [*n* (%)]	459 (21.4%)	48 (14.8%)	0.006
HDL (mmol/L)	1.63 [1.37, 1.96]	1.40 [1.15, 1.13]	<0.001
ALT (U/L)	11.86 [8.69, 17.68]	12.60 [8.85, 18.95]	0.062
AST (U/L)	18.50 [15.00, 22.70]	22.40 [17.60, 29.60]	<0.001
GGT (U/L)	12.00 [9.00, 17.10]	12.10 [8.55, 20.30]	0.612
APOb (g/L)	1.24 ± 0.28^a^	1.20 ± 0.33^a^	0.042
CHOL (mmol/L)	6.29 ± 1.22^a^	5.95 ± 1.58^a^	<0.001
TG (mmol/L)	4.05 ± 1.66^a^	4.00 ± 1.70^a^	0.598
LDL (mmol/L)	3.14 ± 0.94^a^	2.96 ± 1.02^a^	0.002
Glu (mmol/L)	4.88 ± 1.26^a^	4.76 ± 1.12^a^	0.090
CREm (μmol/L)	50.77 ± 11.65^a^	61.53 ± 15.06^a^	<0.001
LDH (U/L)	205.70 [175.60, 255.70]	265.50[218.75,348.90]	<0.001
URIC (μmol/L)	369.65 ± 95.17^a^	462.57 ± 117.44^a^	<0.001
PT (s)	11.15 ± 0.91^a^	11.14 ± 0.92^a^	0.885
FDP (mg/L)	11.15 [7.71, 17.32]	14.80 [9.99, 25.14]	<0.001
TT (s)	16.31 ± 1.12^a^	16.75 ± 1.14^a^	<0.001
D-dimer (mg/L FEU)	3.61 [2.37, 5.25]	4.33 [2.72, 7.22]	<0.001
Fib (g/L)	4.30 ± 0.89^a^	3.85 ± 1.09^a^	<0.001
INR	0.95 ± 0.06^a^	0.95 ± 0.07^a^	0.912
APTT (s)	27.48 ± 3.78^a^	28.58 ± 4.04^a^	<0.001
PLT (× 10^9^/L)	197.09 ± 57.70^a^	178.89 ± 56.77^a^	<0.001
RBC (× 10^12^/L)	3.78 ± 0.50^a^	3.69 ± 0.52^a^	0.001
HCT (%)	33.21 ± 4.32^a^	32.67 ± 4.05^a^	0.033
PDW (fL)	12.92 ± 2.55^a^	14.02 ± 2.88^a^	<0.001
MPV (fL)	10.79 ± 1.02^a^	11.30 ± 1.03^a^	<0.001

*^a^Mean and standard deviation;*

*BMI, body mass index; IVF-ET, in vitro fertilization- embryo transfer; GDM, gestational diabetes mellitus; PROM, premature rupture of membranes; PPROM, preterm premature rupture of membranes; HDL, high-density lipoprotein; AST, aspartame aminotransferase; ALT, alanine aminotransferase; GGT, transglutaminase; APOb, apolipoprotein b; CHOL, cholesterol; TG, triglyceride; LDL, low-density lipoprotein; Glu, glucose; CREm, creatinine; LDH, lactate dehydrogenase; URIC, uric acid; PT, prothrombin time, FDP, fibrinogen degradation products, TT, thrombin time; Fib, fibrinogen; INR, international normalized ration, aPTT, activated partial thromboplastin time; PLT, platelets; RBC, red blood cell count; Hct, hematocrit; PDW, platelet distribution weight, MPV, mean platelet volume.*

### Predictors of Preeclampsia in Twin Pregnancies

**FIGURE 2:**
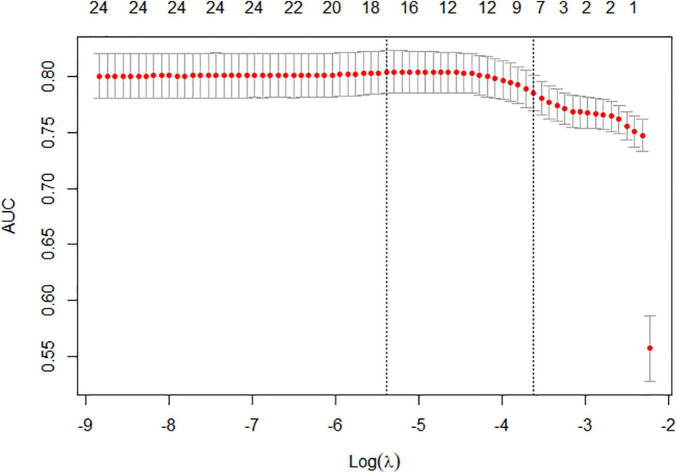
Tuning parameter (lambda) selection in the LASSO model used 10-fold cross-validation *via* 1se criteria for determining the risk of preeclampsia in twin pregnancies. LASSO, Least absolute shrinkage and selection operator.

**FIGURE 3:**
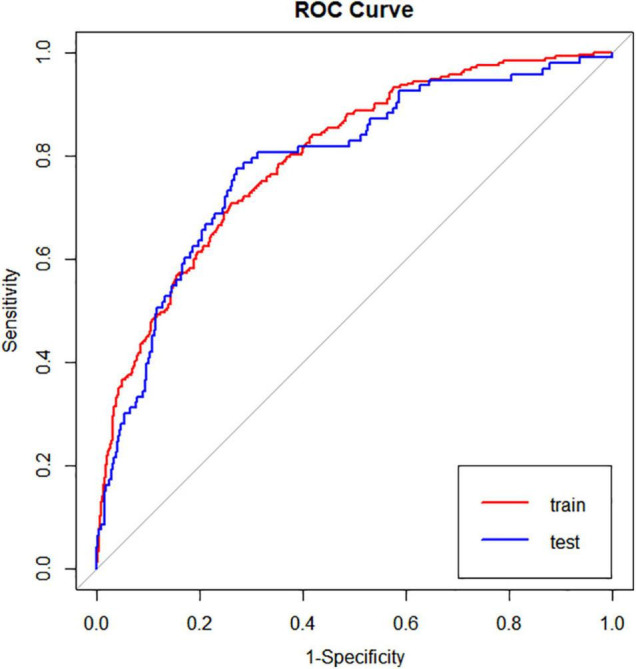
The ROC curve of the preliminary screened variables in the training set and the test set. ROC, receiver operating curve.

We used LASSO regression to screen the peripheral blood parameter variables *via* 1se criteria (λ = 0.02671711) in the training set, and finally seven high-risk factors (CREm, URIC, MPV, HDL, AST, LDH, and Fib) were included in the prediction model (Figure 2). The seven variables (CREm, URIC, MPV, HDL, AST, LDH, and Fib) initially screened by the LASSO regression model have an area under the receiver operating characteristic (ROC) curve of 0.7955 in the training cohort and the area under the ROC curve in the test cohort of 0.7868. There is thus good specificity and sensitivity (Figure 3).

The seven variables were included in the multivariate logistic together with clinically significant variables (primipara, regular prenatal visits, and pre-pregnancy BMI). Multivariate logistic regression analysis of the selected variables showed that primipara [odds ratio (OR): 1.343; 95% confidence interval (CI): 1.024–1.762], pre-pregnancy BMI (OR: 1.071; 95% CI: 1.030–1.114), regular prenatal visits (OR: 0.626; 95% CI: 0.466–0.842), CREm (OR: 1.033; 95% CI: 1.022–1.044), URIC (OR: 1.004; 95% CI: 1.003–1.006), MPV (OR: 1.275; 95% CI: 1.119–1.453), LDH (OR: 1.004; 95% CI: 1.002–1.005), HDL (OR: 0.472; 95% CI: 0.388–0.567), and Fib (OR: 0.794; 95% CI: 0.688–0.916) were independently associated with preeclampsia in twin pregnancies (Table 3).

**TABLE 3:** Multivariate logistic regression analysis of variables to identify factors predictive of preeclampsia in twin pregnancies.

Co-variable	Odds ratio (OR)	95% CI of OR		*P*-value
		Lower Limit	Upper limit	
Primipara	1.343	1.024	1.762	0.033
Regular prenatal visits	0.626	0.466	0.842	0.002
Pre-pregnancy BMI	1.071	1.03	1.114	0.001
CREm	1.033	1.022	1.044	<0.001
URIC	1.004	1.003	1.006	<0.001
MPV	1.275	1.119	1.453	<0.001
HDL	0.472	0.388	0.576	<0.001
AST	1.006	0.996	1.017	0.256
LDH	1.004	1.002	1.005	<0.001
Fib	0.794	0.688	0.916	0.002

*CI, confidence intervals; BMI, body mass index; CREm, creatinine; URIC, uric acid; MPV, mean platelet volume; HDL, high-density lipoprotein; AST, aspartame aminotransferase; LDH, lactate dehydrogenase; Fib, fibrinogen.*

**FIGURE 4:**
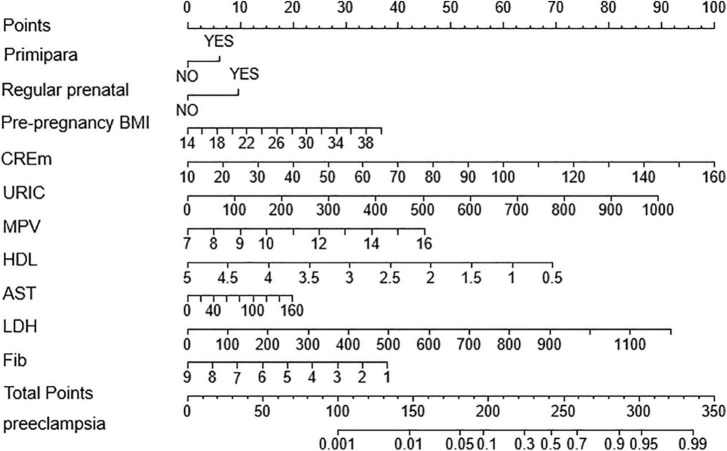
Nomogram for predicting the risk of preeclampsia in twin pregnancies.

To facilitate clinical evaluation and application, we use R’s lrm package to draw the nomogram of the multi-factor logistics regression model. The nomogram provides a practical tool to identify the risk of developing preeclampsia in pregnant women with twin pregnancies (Figure 4).

### Nomogram Validation

**FIGURE 5:**
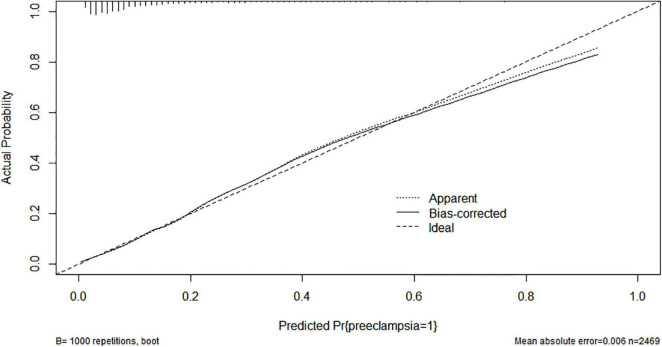
Calibration curves for predicting the risk of preeclampsia in twin pregnancies—nomogram construction (bootstrap = 1 000 repetitions).

After 1,000 Bootstrap self-sampling internal verifications were performed on the model in the data set, this study showed a predictive accuracy of 0.821 (measured by C-index). There was a moderate correlation between the actual outcome and the outcome predicted by the nomogram. This was shown by the calibration plot of the probability of preeclampsia in twin pregnancies (Figure 5).

## Discussion

We found that the probability of twin pregnancy being complicated with preeclampsia was 13.16%, slightly higher than in previous reports (Francisco et al., 2017). We performed a more comprehensive screening of high-risk factors for preeclampsia in twin pregnancies through the acquisition of clinical medical records of patients. The results of logistic multivariate analysis showed that primiparity, regular obstetric visits, pre-pregnancy BMI, CREm, URIC, MPV, HDL, LDH, Fib, primipara, pre-pregnancy BMI, and regular prenatal were independently associated with preeclampsia in twin pregnancies. The nomogram prediction model (concordance index 0.821) was further constructed with good accuracy and conformity, and could make individualized predictions for pregnant women with twin pregnancies complicated by preeclampsia, to allow for timely clinical interventions and thus reduce the number of adverse outcomes in mothers and children.

The expert consensus published by the American College of Obstetricians and Gynecologists showed that primiparity, obesity, advanced age, and assisted reproductive technology were high-risk factors for pregnancies complicated by preeclampsia in singletons [American College of Obstetricians and Gynecologists (ACOG), 2020]. Taguchi et al. (2014) found that pre-pregnancy BMI and primiparity were independent risk factors for preeclampsia in twin pregnancies. Similarly, in another prospective cohort study, Chen et al. (2020) reported that pre-pregnancy BMI was significantly associated with the risk of preeclampsia in twin pregnancies, which is consistent with our findings. In addition, regular antenatal check-ups, strengthening the monitoring of risk factors, and timely intervention can reduce the incidence of preeclampsia in twin pregnancies.

Various biochemical factors in maternal serum play an important role in the pathophysiological development of preeclampsia, and the composition, source, and mechanism are different for each of these factors. At present, the serum markers used to screen for preeclampsia in twin pregnancies mainly include pregnancy-associated plasma protein A, placental protein 13, placental growth factor (Maymon et al., 2017), inhibin A, and unconjugated estriol (Kim et al., 2019); however, widespread roll-out of these indicators is difficult to develop and promote in underdeveloped areas. Therefore, this study measures peripheral blood parameters, screens out predictive indicators with high sensitivity and specificity, and provides a basis for the development of economical and efficient screening programs for twin pregnancies with preeclampsia.

Previous studies on the relationship between MPV and preeclampsia were based on pregnant women with singleton pregnancies, while there were fewer studies involving those with twin pregnancies. In a longitudinal study, Mayer-Pickel et al. (2021) found that MPV is significantly elevated even in early pregnancy in women who develop preeclampsia. A cross-sectional comparative study by Tesfay et al. (2019) showed that MPV had a good predictive value for the development of preeclampsia. Jakobsen et al. (2019) found that women with preeclampsia had significantly higher MPV than in women without preeclampsia in a systematic review and meta-analysis and they believed that MPV is the most promising biomarker for evaluating platelet function in preeclampsia. However, these studies were based on singleton pregnancies; we found that an increase in peripheral blood MPV was associated with an increased risk of preeclampsia in twin pregnancies as well. The increase in MPV reflects the enhancement of platelet activation. In patients with preeclampsia, because of the increase in platelet consumption and destruction, the bone marrow produces and releases a large quantity of platelets, which leads to an increase in MPV (Kaito et al., 2005). Conversely, preeclampsia may cause complex diseases in the endogenous blood coagulation pathway and consumes Fib, resulting in a decrease in Fib (Han et al., 2014). We found an increase in the risk of preeclampsia in twin pregnancies with decreasing Fib, which is similar to the results reported in related studies (Liu et al., 2020).

Some studies have shown that, as pregnancy progresses, serum total cholesterol, TG, and LDL concentrations increase, and this abnormal dyslipidemia during pregnancy is related to preeclampsia and other adverse pregnancy outcomes (Jin et al., 2016). The pathophysiological basis may be that the increase in circulating lipid levels leads to an accumulation of lipids in endothelial cells, which reduces the release of prostacyclin and leads to oxidative stress (Ghio et al., 2011). A meta-analysis by Spracklen et al. (2014) showed that low levels of HDL are significantly associated with the risk of preeclampsia, and our study found that women with twin pregnancies who have low HDL also have this risk.

The main energy supply pathway to the placenta occurs through glycolysis. LDH is an intracellular enzyme required for glycolysis. Under hypoxic conditions, LDH is activated to promote glycolysis and produce large amounts of lactic acid. Therefore, elevated LDH levels often indicate cell damage and dysfunction. Studies have shown that peripheral blood LDH levels in patients with preeclampsia are significantly increased (Wu et al., 2021). Our study found this feature in twin pregnancies with preeclampsia. In addition, some scholars have found that high levels of LDH are significantly related to the occurrence of adverse perinatal outcomes in patients with preeclampsia (Dave et al., 2016). It is necessary to explore in future studies whether there is a dose-response relationship between high levels of LDH and the occurrence of adverse preeclampsia outcomes.

The kidney is an important organ involved in preeclampsia. Damage to glomerular endothelial cells and destruction of the relationship between endothelial cells and podocytes are the main underlying pathogenetic mechanisms of preeclampsia (Moghaddas Sani et al., 2019). Patients with preeclampsia are prone to renal dysfunction caused by extensive renal arteriolar spasm. This causes glomerular swelling, decreased glomerular filtration rate, and decreased renal blood flow, which leads to obstruction of the excretion and clearance of renal metabolites such as URIC and CREm; this results in an increase in serum URIC and CREm. Zhao et al. (2021) found that elevated serum uric acid in Chinese Han women with gestational hypertension indicated an increased risk of their progression to preeclampsia. In a prospective case-control study, Enaruna et al. (2014) found that the URIC level of patients with preeclampsia was significantly increased and that higher levels reflected disease severity. Jhee et al. (2019) developed models using machine learning to predict late-onset preeclampsia and found that CREm levels were one of the most influential variables included in the prediction models. However, Few studies focused on the relationship between these indicators and preeclampsia in twin pregnancy. Our study make up for this and demonstrating that URIC and CREm levels were independent risk factors for preeclampsia in twin pregnancies.

We are aware of the limitations of our study. Our study is a single-center retrospective cohort study, which may result in a risk of overestimating model performance. However, as a tertiary hospital in Southeast China, our number of cases is still representative, and we believe the model would be useful in pregnancy supervision. Further prospective cohort and multi-center joint research will help to further expand the research results, improve the prediction accuracy of the model, and confirm the conclusions.

In summary, the prediction model of preeclampsia in twin pregnancies constructed in this study has good accuracy and high clinical application value, and can be used as a reference for obstetricians. We should pay attention to twin pregnancies with high-risk factors in clinical practice and perform careful screening, early intervention, and active treatment to reduce the occurrence of adverse pregnancy outcomes.

## Conclusion

This study performed a more comprehensive screening of high-risk factors for preeclampsia in twin pregnancies through the acquisition of clinical medical records of patients. The model for the prediction of preeclampsia in twin pregnancies has high accuracy and specificity. It can be used to assess the risk of preeclampsia in twin pregnancies.

## Data Availability Statement

The original contributions presented in the study are included in the article/supplementary material, further inquiries can be directed to the corresponding authors.

## Ethics Statement

The studies involving human participants were reviewed and approved by Ethics Committee of Fujian Maternity and Child Health Hospital. The patients/participants provided their written informed consent to participate in this study.

## Author Contributions

QH and JY: conception and design of the research. SZ and RC: acquisition of data. HZ, SZ, and RC: analysis and interpretation of the data and statistical analysis. QH, JY, and HZ: funding acquisition. QH and SZ: writing of the manuscript. JY, HZ, and QH: critical revision of the manuscript for intellectual content. All authors read and approved the final draft.

## Conflict of Interest

The authors declare that the research was conducted in the absence of any commercial or financial relationships that could be construed as a potential conflict of interest.

## Publisher’s Note

All claims expressed in this article are solely those of the authors and do not necessarily represent those of their affiliated organizations, or those of the publisher, the editors and the reviewers. Any product that may be evaluated in this article, or claim that may be made by its manufacturer, is not guaranteed or endorsed by the publisher.
